# Mean-Variance QTL Mapping Identifies Novel QTL for Circadian Activity and Exploratory Behavior in Mice

**DOI:** 10.1534/g3.118.200194

**Published:** 2018-11-13

**Authors:** Robert W. Corty, Vivek Kumar, Lisa M. Tarantino, Joseph S. Takahashi, William Valdar

**Affiliations:** * Department of Genetics; † Bioinformatics and Computational Biology Curriculum; ‡ The Jackson Laboratory; § Howard Hughes Medical Institute, Department of Neuroscience, University of Texas Southwestern Medical Center, Dallas, TX 75390; ** Lineberger Comprehensive Cancer Center, University of North Carolina at Chapel Hill, Chapel Hill, NC 27599

**Keywords:** variance heterogeneirty, DGLM, mQTL, vQTL, mvQTL

## Abstract

We illustrate, through two case studies, that “mean-variance QTL mapping”—QTL mapping that models effects on the mean and the variance simultaneously—can discover QTL that traditional interval mapping cannot. Mean-variance QTL mapping is based on the double generalized linear model, which extends the standard linear model used in interval mapping by incorporating not only a set of genetic and covariate effects for mean but also set of such effects for the residual variance. Its potential for use in QTL mapping has been described previously, but it remains underutilized, with certain key advantages undemonstrated until now. In the first case study, a reduced complexity intercross of C57BL/6J and C57BL/6N mice examining circadian behavior, our reanalysis detected a mean-controlling QTL for circadian wheel running activity that interval mapping did not; mean-variance QTL mapping was more powerful than interval mapping at the QTL because it accounted for the fact that mice homozygous for the C57BL/6N allele had less residual variance than other mice. In the second case study, an intercross between C57BL/6J and C58/J mice examining anxiety-like behaviors, our reanalysis detected a variance-controlling QTL for rearing behavior; interval mapping did not identify this QTL because it does not target variance QTL. We believe that the results of these reanalyses, which in other respects largely replicated the original findings, support the use of mean-variance QTL mapping as standard practice.

Over the last 25 years, quantitative trait locus (QTL) mapping based on the assumption of homogeneous residual variance and its associated statistical methods (Lander and Botstein 1989; Martínez and Curnow 1992; Haley and Knott 1992; Churchill and Doerge 1994) have successfully identified many QTL that influence the mean value of important complex traits. It is well-appreciated, however, that this approach fails to capture all the complexities of the relationship between genotype and phenotype, and recent interest has expanded the scope of genetic analysis to also look for QTL that cause the residual variance to be higher (or lower) in some individuals than others (Deng and Pare 2011; Rönnegård and Valdar 2012; Shen *et al.* 2012; Geiler-Samerotte *et al.* 2013; Nelson *et al.* 2013; Ayroles *et al.* 2015; Forsberg *et al.* 2015). It has also been noted that modeling QTL effects on the mean, to be effective, can sometimes require accounting for sources of variance heterogeneity—for example, genotype uncertainty (Xu 1998; Feenstra *et al.* 2006), X-chromosome inactivation (Ma *et al.* 2015), and background genetic or environmental factors more generally (Corty and Valdar 2018a)—suggesting that joint models of mean and variance could make QTL mapping more powerful, both for detecting mean-controlling QTL (mQTL) and variance-controlling QTL (vQTL).

Several such methods incorporating joint models of mean and variance have been developed in this context. Two closely related efforts have been the application of the double generalized linear model (DGLM; Smyth 1989) to QTL mapping (Rönnegård and Valdar 2011) and the omnibus test of Cao *et al.* (2014) for genetic association. [See also refs in Rönnegård and Valdar (2012), and recent developments of Soave and Sun (2017); Dumitrascu *et al.* (2018).] Both of these approaches can detect mQTL, vQTL, and QTL influencing some combination of phenotype mean and variance (mvQTL) (Corty and Valdar 2018a). Yet despite the potential of these methods to detect QTL traditional methods overlook, they remain underutilized.

Barriers to widespread adoption include a lack of proven potential in real data applications, as well as the absence of software that is interoperable with existing infrastructure. Apart from those barriers, one reasonable concern is that a novel approach might fail to identify known QTL, adding needless complexity to the interpretation of already-reported QTL. This concern should be largely allayed by the nature of the DGLM as an extension of the linear model, simplifying to the latter when variance heterogeneity is absent [with similar arguments holding for the omnibus test of Cao *et al.* (2014)]. In fact, rather than add complexity, the DGLM automatically classifies QTL into mQTL, vQTL, or mvQTL, clarifying the genotype-phenotype relationship.

Here we demonstrate, with two real data examples available from the Mouse Phenome Database (Bogue *et al.* 2017), that QTL mapping using the DGLM, which we term “mean-variance QTL mapping”, largely replicates the results of standard QTL mapping and detects additional QTL that the traditional analysis does not. In two companion articles, we demonstrate typical usage of R package vqtl, which implements mean-variance QTL mapping (Corty and Valdar 2018b), and describe the how mean-variance QTL mapping and its associated permutation procedures reliably detects QTL in the face of variance heterogeneity arising from background factors (*i.e.*, genetic or non-genetic factors outside the targeted QTL) (Corty and Valdar 2018a).

## Statistical Methods

### Traditional QTL mapping based on the standard linear model (SLM)

The traditional approach to mapping a quantitative trait in an experimental cross with no population structure (*e.g.*, an F2 intercross or backcross) involves fitting, at each locus in turn, a linear model of the following form. Letting $y_{i}$ denote the phenotype value of individual *i*, this phenotype is modeled as

$$y_{i}∼\text{N}\left(m_{i},\;\sigma^{2}\right),$$

where $\sigma^{2}$ is the residual variance, and the expected phenotype mean, $m_{i},$ is predicted by effects of QTL genotype and, optionally, effects of covariates. In the reanalyses performed here, $m_{i}$ is modeled to include a covariate of sex and additive and dominance effects of QTL genotype, that is,

$$m_{i}=\mu +{\text{sex}}_{i}\beta_{\text{sex}}+a_{i}\beta_{a}+d_{i}\beta_{d},$$

where *μ* is the intercept, $\beta_{\text{sex}}$ is the sex effect, with ${\text{sex}}_{i}$ indicating (0 or 1) the sex of individual *i*, and $\beta_{a}$ and $\beta_{d}$ are the additive and dominance effects of a QTL whose genotype is represented by $a_{i}$ and $d_{i}$ defined as follows: when QTL genotype is known, $a_{i}$ is the count (0,1,2) of one parental allele, and $d_{i}$ indicates heterozygosity (0 or 1); when QTL genotype is inferred based on flanking marker data, as is done here, $a_{i}$ and $d_{i}$ are replaced by their corresponding probabilistic expectations (Haley and Knott 1992; Martínez and Curnow 1992). The evidence for association at a given putative QTL is based on a comparison of the fit of the model above with that of a null model that is identical except for the QTL effects being omitted. These models and their comparison we henceforth refer to as the standard linear model (SLM) approach.

### Mean-variance QTL mapping based on the double generalized linear model (DGLM)

The statistical model underlying mean-variance QTL mapping, the double generalized linear model (DGLM; Smyth 1989 and Rönnegård and Valdar 2011), elaborates the SLM approach by modeling a potentially unique value of $\sigma^{2}$ for each individual, as

$$y_{i}∼\text{N}\left(m_{i},\;\sigma_{i}^{2}\right),$$

where $m_{i}$ has the same meaning as in the SLM, but now $\sigma_{i}^{2}$ is linked to its own linear predictor $v_{i}$ as

$$\sigma_{i}=\text{exp}\left(v_{i}\right),$$

where the exponentiation ensures that $\sigma_{i}$ is always positive, though $v_{i}$ is unconstrained. The linear predictors for $m_{i}$ and $v_{i}$ are modeled as

$$\text{mean}:\;m_{i}=\mu +{\text{sex}}_{i}\beta_{\text{sex}}+a_{i}\beta_{a}+d_{i}\beta_{d}$$ (1)

$$\text{log}(\text{variance}):\;v_{i}=\mu_{v}+{\text{sex}}_{i}\gamma_{\text{sex}}+a_{i}\gamma_{a}+d_{i}\gamma_{d}$$

where *μ*, $a_{i},$ $d_{i}$, ${\text{sex}}_{i},$ and the *β*’s are as before, $\mu_{v}$ is an intercept representing the (log of the) “baseline” residual variance, and $\gamma_{a},$ $\gamma_{d},$ and $\gamma_{\text{sex}}$ are the effects of the QTL and covariates on $v_{i}.$

The evidence for a QTL association is now defined through three distinct model comparisons, corresponding to testing for an mQTL, a vQTL, or an mvQTL. In each case, the fit of the “full” model in Equation 1 is compared with that of a different fitted null: for the mQTL test, the null model omits the QTL effects on the mean (*i.e.*, $\beta_{a}=\beta_{d}=0$); for the vQTL test, the null model omits the QTL effects on the variance (*i.e.*, $\gamma_{a}=\gamma_{d}=0$); and for the mvQTL test, the null model omits QTL effects on both mean and variance (*i.e.*, $\beta_{a}=\beta_{d}=\gamma_{a}=\gamma_{d}=0$). These tests are detailed in Corty and Valdar (2018a).

### Genomewide significance and FWER-adjusted p-values

The model comparisons described above constitute the SLM test and the three DGLM-based tests and each produces a likelihood ratio (LR) statistic. These LR statistics are converted to *p*-values that are adjusted for the family-wise error rate (FWER) across loci, *i.e.*, *p*-values on the scale of genomewide significance. This adjustment is performed separately for each test by calculating an empirical distribution for the LR statistic under permutation, much in the spirit of Churchill and Doerge (1994) but with some modifications, namely that different tests have differently structured permutations. Briefly, let $G_{i}$ be the full set of genetic information for individual *i*, that is, the genotypes or genotype probabilities across all loci. For the SLM and mvQTL tests, we define a permutation as randomly shuffling the $G_{i}$’s across individuals; for the mQTL test, the permutations apply this shuffle only to the genotype information in the full model’s mean component; for the vQTL test, the permutations apply the shuffle only to the genotype information in the full model’s variance component. For a given test, for each permutation we calculate LR statistics across the genome and record the maximum; the maxima of over all permutations is fitted to a generalized extreme value distribution, and the upper tail probabilities of this fitted distribution are used to calculated the FWER-adjusted *p*-values for the LR statistics in the unpermuted data [see Dudbridge and Koeleman (2004), and, *e.g.*, Valdar *et al.* 2006; more details in Corty and Valdar (2018a)]. An FWER-adjusted *p*-value can be interpreted straightforwardly: it is the probability of observing an association statistic this large or larger in a genome scan of a phenotype with no true associations.

### Data Availability

All data and scripts used to conduct the analyses presented here and plot results are archived in a public, static repository at with DOI: 10.5281/zenodo.1453905. Specifically, the raw data files are:

- 1_Kumar2014.csv The phenotype and genotype data from Kumar *et al.* (2013) that was reanalyzed. This dataset is also available from the Mouse Phenome Database (Bogue *et al.* 2017) at https://phenome.jax.org/projects/Kumar1.
- 4_Bailey2008.csv The phenotype and genotype data from Bailey *et al.* (2008) that was reanalyzed. This dataset is also available from the Mouse Phenome Database at https://phenome.jax.org/projects/Bailey1.
- 9_actogram_data The raw data on circadian activity from Kumar *et al.* (2013) that was used to plot actograms

The analysis and plotting scripts are:

- 2_run_Kumar_scans.R This script runs genome scans with R/qtl and R/vqtl on the data from Kumar *et al.* (2013).
- 3_plot_Kumar_scans.R This script plots the results of the reanalysis of Kumar *et al.* (2013).
- 5_run_Bailey_scans.R This script runs genome scans with R/qtl and R/vqtl on the data from Bailey *et al.* (2008).
- 6_plot_Bailey_scans.R This script plots the results of the reanalysis of Bailey *et al.* (2008).
- 7_prune_big_files.R This script strips out redundant information from the results to make the file size smaller to share more easily online.
- 8_power_simulations.R This script runs the power simulation comparing the DGLM to the SLM at the QTL identified in the Kumar reanalysis.

**Figure 1:**
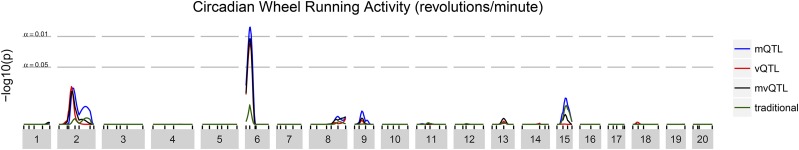
Genome scan for Kumar *et al.* circadian wheel running activity. The horizontal axis shows chromosomal location and the vertical axis shows FWER-controlling *p*-values for the association between each genomic locus and circadian wheel running activity. Line colors indicate the test type, with traditional (green) representing the traditional SLM-based QTL association, and the mQTL (blue), vQTL (red) and mvQTL (black) tests calculated using (the DGLM-based) mean-variance QTL mapping.

**Figure 2:**
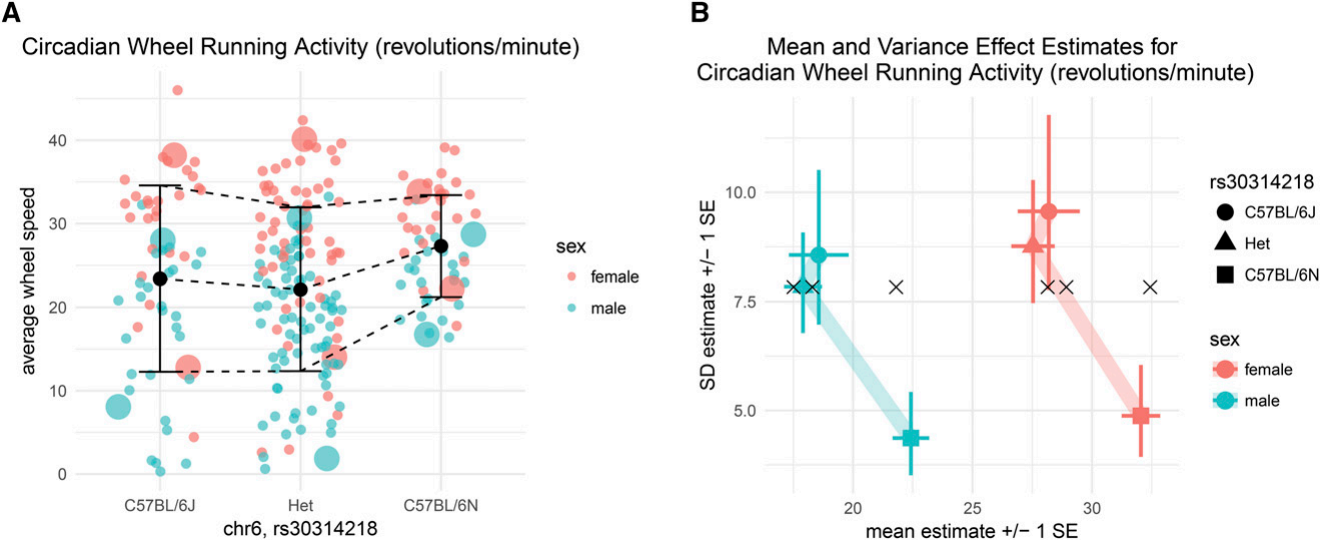
(a) Average wheel speed (revolutions/minute) of all mice. It is visually apparent that female mice had higher circadian wheel running activity than male mice and that mice that homozygous for C57BL/6N had higher circadian wheel running activity and less intra-genotype variation. Large dots indicate the mice whose activity is shown in actogram form (males in Figure 3; all in Figure S2). (b) Predicted mean and variance of mice according to sex and allele at the QTL. What was visually apparent in (a) is captured by the DGLM. The estimated parameters relating to mice that are homozygous for the C57BL/6N allele imply a higher expected value and a lower residual variance than the other two genotype groups. Black x’s indicate the estimates from the SLM, very similar to the DGLM estimates in the horizontal (mean) axis, but homogeneous in the vertical (variance) axis.

The results of running the analysis and plotting scripts are:

- Kumar_scans_1000_perms.RDS This file contains the results of the reanalysis of Kumar *et al.* (2013).
- Bailey_scans_1000_perms.RDS This file contains the results of the reanalysis of Bailey *et al.* (2008).
- Kumar_plots This directory contains the figures generated by 3_plot_Kumar_scans.R (Figures 1, 2, and S1).
- Bailey_plots This directory contains the figures generated by 6_plot_Bailey_scans.R (Figures 4, 5, S5, S6, S7, and S8).

Supplemental material available at Figshare: https://doi.org/10.25387/g3.7284575.

## Reanalysis of kumar et al. reveals a new mqtl for circadian wheel running activity

### Summary of Original Study

Kumar *et al.* (2013) intercrossed C57BL/6J and C57BL/6N, two closely-related strains of C57BL6 that diverged in 1951, approximately 330 generations ago. Due to recent coancestry of the parental strains, this cross is termed a “reduced complexity cross”, and their limited genetic differences ensure that any identified QTL region can be narrowed to a small set of variants bioinformatically. The intercross resulted in 244 F2 offspring, 113 female and 131 male, which were tested for acute locomotor response to cocaine (20mg/kg) in the open field. One to three weeks following psychostimulant response testing, the mice were tested for circadian wheel running activity.

Analysis of wheel running data were carried out using ClockLab software v6.0.36. For calculation of activity, 20 day-epoch in DD was used in order to have standard display between actograms. Analysis of other circadian measures such as period (tau) or amplitude were carried out using methods previously described (Shimomura *et al.* 2001). All animal protocols were approved by the Institutional Animal Care and Use Committee (IACUC) of the University of Texas Southwestern Medical Center

Traditional QTL mapping with the SLM, reported in Kumar *et al.* (2013), detected a single large-effect QTL for cocaine-response traits on chromosome 11, but no QTL for circadian activity. A later study by another group nonetheless observed that the circadian activity of the two strains showed significant differences (Banks *et al.* 2015).

### Reanalysis with traditional QTL mapping and mean-variance QTL mapping

For the cocaine response traits, traditional QTL mapping and mean-variance QTL mapping gave results that were nearly identical to the originally-published analysis in Kumar *et al.* (2013) (Figure S1).

For the circadian wheel running activity trait, however, traditional QTL mapping identified no QTL (Figure 1 in green) but mean-variance QTL mapping identified one QTL on chromosome 6 (Figure 1 in blue, black, and red). In this case, all three tests were statistically significant, but the most significant was the mQTL test (blue), so we discuss it as an mQTL. The most significant genetic marker was rs30314218 on chromosome 6, at 18.83 cM, 40.0 Mb, with a FWER-controlling *p*-value of 0.0063. The mQTL explains 8.4% of total phenotype variance by the traditional definition of percent variance explained (*e.g.*, Broman and Sen 2009).

### Understanding the Novel QTL

Though they test for the same pattern, the mQTL test of mean-variance QTL mapping identified a QTL where the traditional QTL test did not. This discordance may arise when there is variance heterogeneity in the mapping population. In this case, mice homozygous for the C57BL/6N allele at the mQTL have both higher average wheel running activity and lower residual variance in wheel running activity than mice with other genotypes (Figure 2a).

**Figure 3:**
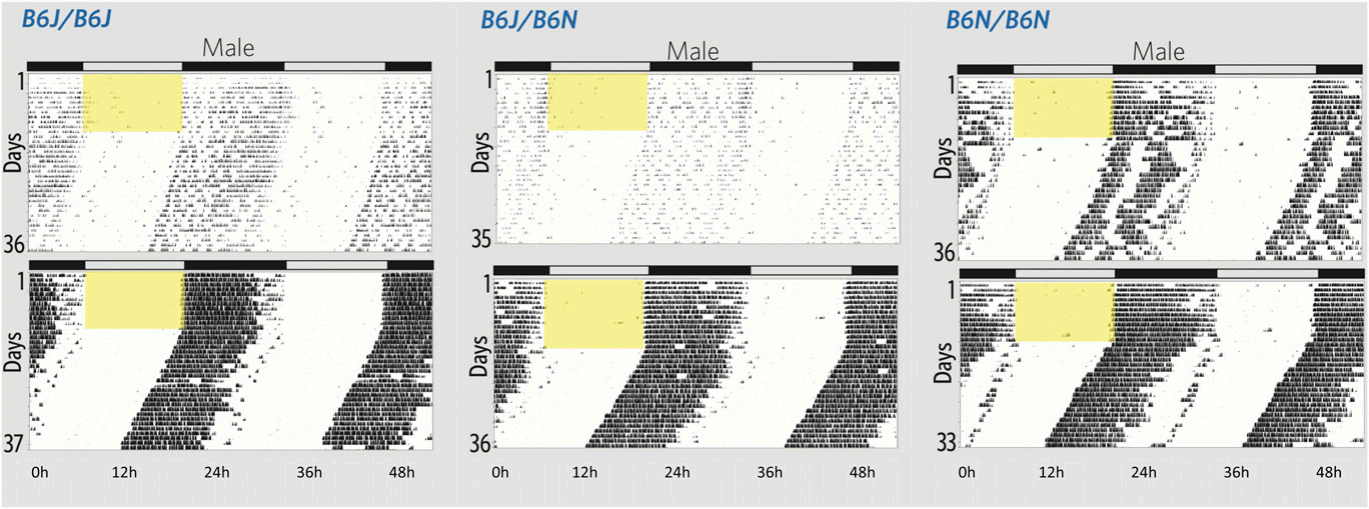
Double-plotted actograms illustrate the variation in wheel running activity of male mice based on their genotype at rs30314218. On reading a single actogram: An actogram illustrates the activity of a single mouse over the course of an experiment. Each day of the experiment is represented by a histogram, with bin width of six minutes. Histograms are stacked vertically. Additionally, each day is shown twice (repeated horizontally) so that there is no time of day that is illegible due to the plot edges. Yellow box indicates when lights were on. On reading this six-actogram plot: Recall that the DGLM estimates a unique mean and standard deviation (SD) for each genotype. The mice whose actograms are shown here had an activity level that is one genotype-specific SD greater than (top) or less than (bottom) the genotype-specific mean. The difference between the two is much less in the C57BL/6N homozygotes than in the other genotypes, reflecting the decreased phenotype variance among C57BL/6N homozygotes. The animals shown in this figure are marked with large blue circles in Figure 2a. A larger figure that also includes female mice as well as the ID’s of all plotted mice are in the supplement (Figure S2 and Table 2).

The identification of this QTL by mean-variance QTL mapping but not traditional QTL mapping can be understood by contrasting how the DGLM and SLM fit the data at this locus.

For the SLM, a single value of the residual standard deviation *σ* is estimated for all mice. Approximately 25% of the mice are homozygous for the C57BL/6N allele, so *σ* is estimated mostly based on heterozygous mice and homozygous C57BL/6J mice. The SLM estimates $\hat{\sigma }=7.83$, a slight underestimate for some genotype-sex combinations, and a drastic overestimate for the homozygous C57BL/6N of both sexes (Figure 2b). With *σ* overestimated for the C57BL/6N homozygotes, the addition of a locus effect to the null model results in only a limited increase in the likelihood, one that could reasonably be caused by chance alone. For the DGLM, six different values of *σ* are estimated, one for each genotype-sex combination (Figure 2b). With a better-estimated (lower) $\hat{\sigma }$ for the C57BL/6N homozygotes, the addition of the locus effect to the null model results in a greater increase in the likelihood, one that is very unlikely due to chance alone.

A simulation based on the estimated coefficients shows that at a false positive rate of $5\times 10^{-4}$, relevant for genome-wide significance testing, the SLM has 61% power to reject the null at this locus and the DGLM has 90% power (See file 8_power_simulations.R).

### Variant Prioritization

Reduced complexity crosses allow variant prioritization to proceed quickly because of the number of segregating variants is small. Using 1000 nonparametric bootstrap resamples, the QTL interval was estimated as 13.5-23.5 cM (90% CI), which translates to physical positions of 32.5 - 48.5 Mb using Mouse Map Converter’s sex averaged Cox map (Cox *et al.* 2009). Since this interval contains no genes or previously identified QTL shown to regulate circadian rhythms, we prioritized candidates by identifying variants between C57BL/6J and C57BL/6NJ based on Sanger mouse genome database (Keane *et al.* 2011; Simon *et al.* 2013), which yielded 463 SNPs, 124 indels, and 3 structural variants (Table 1).

**Table 1:** Genetic Variants in QTL interval for circadian wheel running activity

location	indel	SNP	SV	Total
exon, missense	–	2	–	2
intron	58	247	–	305
3′ UTR	–	3	–	3
upstream	6	29	–	35
downstream	7	20	–	27
intergenic	53	161	–	214
unclassified	–	1	3	4

**Table 2:** The characteristics of the mice plotted in Figure 3

genotype at rs30314218	sex	activity in the DD (rev/min)
6J	female	12.79
6J	female	38.20
6J	male	8.07
6J	male	27.99
Het	female	14.03
Het	female	40.13
Het	male	1.87
Het	male	30.68
6N	female	22.22
6N	female	33.85
6N	male	16.75
6N	male	28.71

Of these variants, none of the indels or structural variants were nonsynonymous. Two SNPs were predicted to lead to missense changes (T to A at position 6: 39400456 in *Mkrn1*, and A to A/C at 6:48486716 in *Sspo*). The variant in *Sspo* was a very low confidence call and therefore likely a false positive.

The *Mkrn1* (makorin ring finger protein 1) variant is a mutation in C57BL/6J that changes a highly conserved (Figure S3 and Figure S4) tyrosine to asparagine with rsID rs30899669. It was determined to be the best candidate variant in the QTL interval. The Mkrn1 protein is a ubiquitin E3 ligase with zinc finger domains with poorly defined function (Kim *et al.* 2005). It is expressed at low levels widely in the brain according to Allen Brain Atlas and EBI Expression Atlas (Lein *et al.* 2007; Kapushesky *et al.* 2009; McWilliam *et al.* 2013). Functional analysis will be necessary to experimentally confirm that this variant in *Mkrn1* is indeed the causative mutation that led, in a dominant fashion, to the decreased expected value and increased variance of circadian wheel running activity observed in mice with at least one copy of the C57BL/6J haplotype in the QTL region in this study.

## Reanalysis of bailey et al. identifies a new vqtl for rearing behavior

### Summary of Original Study

Bailey *et al.* (2008) intercrossed C57BL/6J and C58/J mice, two strains known to be phenotypically similar for anxiety-related behaviors, as a control cross for an ethylnitrosourea mutagenesis mapping study. The intercross resulted in 362 F2 offspring, 196 females and 166 males. Six open-field behaviors were measured at approximately 60 days of age in a 43cm by 43cm by 33cm white arena for ten minutes. All phenotypes were transformed with the rank-based inverse normal transform to limit the influence of outliers. The authors reported 7 QTL spread over five of the six measured traits, but none for rearing behavior.

### Reanalysis with SLM and DGLM

SLM-based QTL analysis replicated the originally-reported LOD curves. Significance thresholds to control FWER at 0.05 were estimated by 10,000 permutations, using the method described in the original publication, but found to be meaningfully higher than the originally-reported thresholds. Of the 7 originally-reported QTL, 3 exceeded the newly-estimated thresholds (Figure S5).

**Figure 4:**
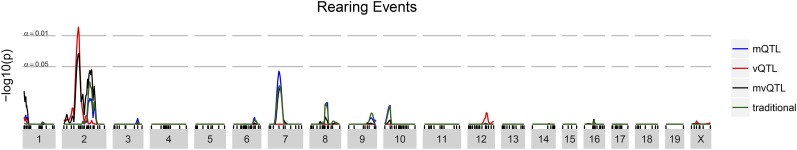
Genome scan for Bailey *et al.* rearing behavior. The x axis shows chromosomal location and the y axis shows FWER-controlling *p*-values for the association between each genomic locus and the Box-Cox transformed rearing behavior.

The DGLM-based reanalysis was initially conducted with the rank-based inverse normal transformed phenotypes, to maximize the comparability with the original study. This reanalysis largely replicated the results of the SLM-based analysis and identified a statistically-significant vQTL for rearing behavior on chromosome 2 (Figure 4 and Figure S5). The top marker under the peak was at 38.6cM and 65.5Mb.

There are well-known and well-founded concerns that inappropriate scaling of phenotypes can produce spurious vQTL (Rönnegård and Valdar 2012; Sun *et al.* 2013; Shen and Ronnegard 2013). Therefore, the rearing phenotype was analyzed under a variety of additional transforms: none, log, square root, and $\frac{1}{4}$^th^ power (the transformation recommended by the Box-Cox procedure). Because the trait is a “count” and a positive mean-variance correlation was observed, the trait was further analyzed with a Poisson double generalized linear model with its canonical link function (log). In all cases, the same genomic region on chromosome 2 was identified as a statistically significant vQTL (p<0.01) (Figure S6, Figure S7 and Figure S8). Though all transformations yielded similar results, we highlight the Box-Cox transformed analysis recommended for transformation selection in Rönnegård and Valdar (2011).

### Understanding the Novel QTL

**Figure 5:**
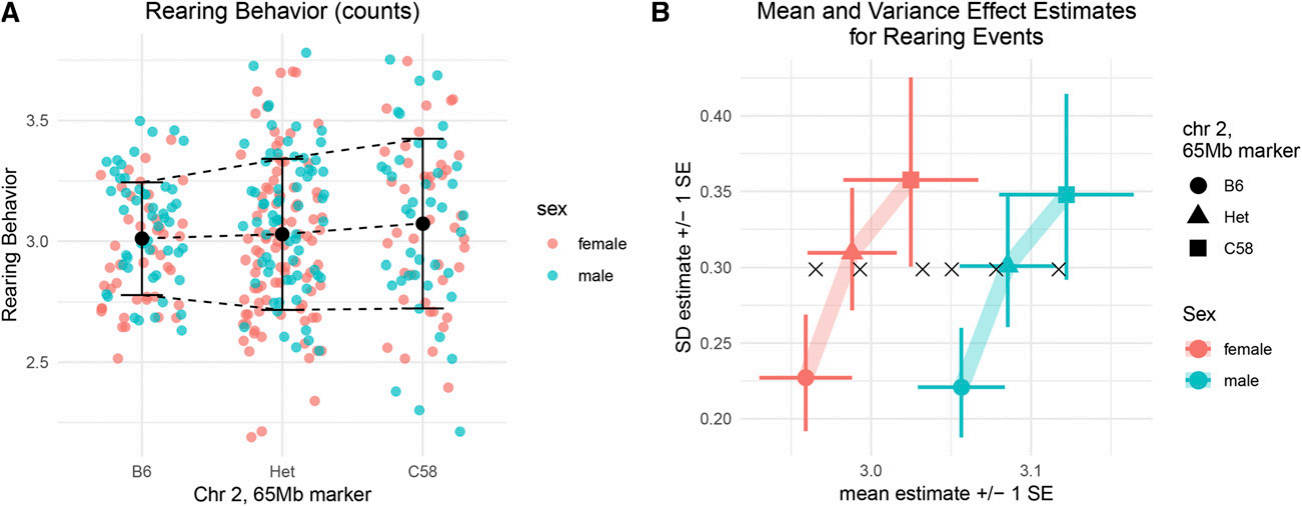
(a) “Total Rearing Events”, transformed by the Box-Cox procedure, stratified by sex and genotype at the top marker. (b) Predicted mean and variance of mice according to sex and allele at the top marker.

In this case, the DGLM-based analysis identified a vQTL, a pattern of variation across genotypes not targeted by traditional, SLM-based, QTL analysis. The phenotype values, when stratified by genotype at the top locus, illustrate clear variance heterogeneity (Figure 5a). The effects and their standard errors estimated by the DGLM fitted at the top locus corroborate the impression from simply viewing the data, that the locus is a vQTL but not an mQTL (Figure 5b).

## Discussion

We have demonstrated through two case studies that mean-variance QTL mapping based on the DGLM expands the range of QTL that can be detected, including both mQTL at loci that exhibit variance heterogeneity and vQTL. In an era where ever more complete and complex data on biological systems is becoming available, this modest elaboration of an existing approach represents a step toward the broader goal of characterizing the wide array of patterns of association between genotype, environment, and phenotype.

In the reanalysis of Kumar *et al.*, mean-variance QTL mapping identified the same QTL as traditional, SLM-based QTL mapping for cocaine response traits and one novel mQTL for a circadian behavior trait. Such an mQTL would likely have been detected by a traditional QTL analysis with a larger mapping population: Through simulation, we estimated that the additional power to detect the mQTL was equivalent to the power increase that would have come from increasing the sample size by ≈100 mice, from 244 to ≈350 (See file 8_power_simulations.R). Given the considerable effort and expense associated with conducting an experimental cross or expanding the size of the mapping population, there seems to be little to be gained by omitting a DGLM-based analysis.

In the reanalysis of Bailey *et al.*, mean-variance QTL mapping identified a novel vQTL for an exploratory behavior. A vQTL such as this would not be detected by the traditional QTL analysis no matter how large the mapping population because the pattern is entirely undetectable by the SLM.

The identification of a vQTL raises important issues related to phenotype transformation and the interpretation of findings, but both are manageable, as we have illustrated here. The criticism that a spurious vQTL can arise as the result of an inappropriate transformation is based on the observation that when genotype means are unequal, there always exists a (potentially exotic) transformation that diminishes the extent of variance heterogeneity (Sun *et al.* 2013). Thus, any other transformation (including none at all) can be seen as inflationary toward variance heterogeneity. In this context, however, an “inappropriate transformation” leads not to the misclassification of a non-QTL as a QTL, but an mQTL as a vQTL.

To the extent that the goal of QTL mapping is to understand a trait’s genetic architecture, this criticism is valid and should be addressed by considering a wide range of transformations, alternative models, and parameterizations. To the extent that the goal of QTL mapping is to identify genomic regions that contain genes and regulatory factors that influence a trait in any way, we argue that such a misclassification is often irrelevant. Whether we pursue bioinformatic follow-up to identify QTN in a region because it was identified as an mQTL or a vQTL need not change our downstream efforts.

In summary, we advocate for the use of mean-variance QTL mapping not as an additional flourish to consider after conducting an SLM-based QTL mapping effort, but rather as a drop-in replacement. This approach should not be too alien — when variance heterogeneity is absent, it simplifies to the well-known SLM-based approach. Full-featured software that implements this approach is described in a companion article (Corty and Valdar 2018b).

Last, we note an additional benefit conferred by mean-variance QTL mapping not discussed in depth here. Variance heterogeneity can also derive from factors acting in the “background”, that is, arising from experimental or biological variables that are outside the main focus of testing but that nonetheless predict phenotypic variability and thereby inform the relative precision of one individual’s phenotype over another. In the case studies presented here, the only background factor considered was sex. But, more generally, any factor that a researcher considers as a potentially important covariate that should be modeled can be included not only as a mean covariate (as with the SLM) but also as a variance covariate. As described in a companion article, Corty and Valdar (2018a), this practice has the potential to both deliver increased power to detect QTL (mQTL, vQTL, and mvQTL) as well as increase the reproducibility of published QTL.

## References

[bib1] Ayroles J. F. Buchanan S. M. O’Leary C. Skutt-Kakaria K. Grenier J. K., 2015 Behavioral idiosyncrasy reveals genetic control of phenotypic variability. Proc. Natl. Acad. Sci. USA 112: 6706–6711. 10.1073/pnas.1503830112 25953335 PMC4450409

[bib2] Bailey J. S. Grabowski-Boase L. Steffy B. M. Wiltshire T. Churchill G. A., 2008 Identification of quantitative trait loci for locomotor activation and anxiety using closely related inbred strains. Genes Brain Behav. 7: 761–769. 10.1111/j.1601-183X.2008.00415.x 19130624 PMC2888940

[bib3] Banks G. Heise I. Starbuck B. Osborne T. Wisby L., 2015 Genetic background influences age-related decline in visual and nonvisual retinal responses, circadian rhythms, and sleep. Neurobiol. Aging 36: 380–393. 10.1016/j.neurobiolaging.2014.07.040 25179226 PMC4270439

[bib4] Bogue M. A. Grubb S. C. Walton D. O. Philip V. M. Kolishovski G., 2017 Mouse phenome database: an integrative database and analysis suite for curated empirical phenotype data from laboratory mice. Nucleic Acids Res. 46: D843–D850. 10.1093/nar/gkx1082 PMC5753241 29136208

[bib5] Broman K. W. Sen S., 2009 A Guide to QTL Mapping with R/qtl, Springer, New York 10.1007/978-0-387-92125-9

[bib6] Cao Y. Wei P. Bailey M. Kauwe J. S. K. Maxwell T. J., 2014 A versatile omnibus test for detecting mean and variance heterogeneity. Genet. Epidemiol. 38: 51–59. 10.1002/gepi.21778 24482837 PMC4019404

[bib7] Churchill G. A. Doerge R. W., 1994 Empirical Threshold Values for Quantitative Trait Mapping. Genetics 138: 963–971. 7851788 10.1093/genetics/138.3.963 PMC1206241

[bib8] Corty R. W. Valdar W., 2018 a Mean-Variance QTL Mapping on a Background of Variance Heterogeneity. G3: Genes, Genomes, Genetics (Bethesda) xxx-xxx. 10.1534/g3.118.200790 PMC6288843 30389794

[bib9] Corty R. W. Valdar W., 2018 b vqtl: An R package for Mean-Variance QTL Mapping. G3: Genes, Genomes, Genetics (Bethesda) xxx-xxx. 10.1534/g3.118.200642 PMC6288833 30389795

[bib10] Cox A. Ackert-Bicknell C. L. Dumont B. L. Ding Y. Bell J. T., 2009 A New Standard Genetic Map for the Laboratory Mouse. Genetics 182: 1335–1344. 10.1534/genetics.109.105486 19535546 PMC2728870

[bib11] Deng W. Q. Pare G., 2011 A fast algorithm to optimize SNP prioritization for gene-gene and gene-environment interactions. Genet. Epidemiol. 35: 729–738. 10.1002/gepi.20624 21922538

[bib12] Dudbridge F. Koeleman B. P. C., 2004 Efficient computation of significance levels for multiple associations in large studies of correlated data, including genomewide association studies. Am. J. Hum. Genet. 75: 424–435. 10.1086/423738 15266393 PMC1182021

[bib13] Dumitrascu B. Darnell G. Ayroles J. Engelhardt B. E., 2018 Statistical tests for detecting variance effects in quantitative trait studies. Bioinformatics. bty565 10.1093/bioinformatics/bty565 PMC6330007 29982387

[bib14] Feenstra B. Skovgaard I. M. Broman K. W., 2006 Mapping quantitative trait loci by an extension of the Haley-Knott regression method using estimating equations. Genetics 173: 2269–2282. 10.1534/genetics.106.058537 16702423 PMC1569686

[bib15] Forsberg S. K. Andreatta M. E. Huang X. Y. Danku J. Salt D. E., 2015 The Multi-allelic Genetic Architecture of a Variance-Heterogeneity Locus for Molybdenum Concentration in Leaves Acts as a Source of Unexplained Additive Genetic Variance. PLoS Genet. 11: e1005648 10.1371/journal.pgen.1005648 26599497 PMC4657900

[bib16] Geiler-Samerotte K. A. Bauer C. R. Li S. Ziv N. Gresham D., 2013 The details in the distributions: Why and how to study phenotypic variability. Curr. Opin. Biotechnol. 24: 752–759. 10.1016/j.copbio.2013.03.010 23566377 PMC3732567

[bib17] Haley C. S. Knott S., 1992 A simple regression method for mapping quantitative trait loci in line crosses using flanking markers. Heredity (Edinb) 69: 315–324. 10.1038/hdy.1992.131 16718932

[bib18] Kapushesky M. Emam I. Holloway E. Kurnosov P. Zorin A., 2009 Gene expression Atlas at the European Bioinformatics Institute. Nucleic Acids Res. 38: D690–D698. 10.1093/nar/gkp936 19906730 PMC2808905

[bib19] Keane T. M. Goodstadt L. Danecek P. White M. A. Wong K., 2011 Mouse genomic variation and its effect on phenotypes and gene regulation. Nature 477: 289–294. 10.1038/nature10413 21921910 PMC3276836

[bib20] Kim J. H. Park S. M. Kang M. R. Oh S. Y. Lee T. H., 2005 Ubiquitin ligase MKRN1 modulates telomere length homeostasis through a proteolysis of hTERT. Genes Dev. 19: 776–781. 10.1101/gad.1289405 15805468 PMC1074314

[bib21] Kumar, V., K. Kim, C. Joseph, S. Kourrich, S.-H. Yoo, *et al.*, 2013 C57BL/6N Mutation in Cytoplasmic FMRP interacting protein 2 Regulates Cocaine Response. Science (80). 342: 1508–1512 10.1126/science.1245503 PMC4500108 24357318

[bib22] Lander E. S. Botstein S., 1989 Mapping mendelian factors underlying quantitative traits using RFLP linkage maps. Genetics 121: 185–199. 2563713 10.1093/genetics/121.1.185 PMC1203601

[bib23] Lein E. S. Hawrylycz M. J. Ao N. Ayres M. Bensinger A., 2007 Genome-wide atlas of gene expression in the adult mouse brain. Nature 445: 168–176. 10.1038/nature05453 17151600

[bib24] Ma L. Hoffman G. Keinan A., 2015 X-inactivation informs variance-based testing for X-linked association of a quantitative trait. BMC Genomics 16: 241 10.1186/s12864-015-1463-y 25880738 PMC4381508

[bib25] Martínez O. Curnow R. N., 1992 Estimating the locations and the sizes of the effects of quantitative trait loci using flanking markers. Theor. Appl. Genet. 85: 480–488. 10.1007/BF00222330 24197463

[bib26] McWilliam H. Li W. Uludag M. Squizzato S. Park Y. M., 2013 Analysis Tool Web Services from the EMBL-EBI. Nucleic Acids Res. 41: W597–W600. 10.1093/nar/gkt376 23671338 PMC3692137

[bib27] Nelson R. M. Pettersson M. E. Carlborg Ö., 2013 A century after Fisher: Time for a new paradigm in quantitative genetics. Trends Genet. 29: 669–676. 10.1016/j.tig.2013.09.006 24161664

[bib28] Rönnegård L. Valdar W., 2011 Detecting major genetic loci controlling phenotypic variability in experimental crosses. Genetics 188: 435–447. 10.1534/genetics.111.127068 21467569 PMC3122324

[bib29] Rönnegård L. Valdar W., 2012 Recent developments in statistical methods for detecting genetic loci affecting phenotypic variability. BMC Genet. 13: 63 10.1186/1471-2156-13-63 22827487 PMC3493319

[bib30] Shen X. Pettersson M. Ronnegard L. Carlborg O., 2012 Inheritance beyond plain heritability: variance-controlling genes in Arabidopsis thaliana. PLoS Genet. 8: e1002839 10.1371/journal.pgen.1002839 22876191 PMC3410891

[bib31] Shen X. Ronnegard L., 2013 Issues with data transformation in genome-wide association studies for phenotypic variability. F1000 Res. 2: 200 10.12688/f1000research.2-200.v1 PMC3869493 24555098

[bib32] Shimomura K. Low-Zeddies S. S. King D. P. Steeves T. D. Whiteley A., 2001 Genome-wide epistatic interaction analysis reveals complex genetic determinants of circadian behavior in mice. Genome Res. 11: 959–980. 10.1101/gr.171601 11381025

[bib33] Simon M. M. Greenaway S. White J. K. Fuchs H. Gailus-Durner V., 2013 A comparative phenotypic and genomic analysis of c57bl/6j and c57bl/6n mouse strains. Genome Biol. 14: R82 10.1186/gb-2013-14-7-r82 23902802 PMC4053787

[bib34] Smyth G. K., 1989 Generalized linear models with varying dispersion. J. R. Stat. Soc. Ser. B Methodol. 51: 47–60.

[bib35] Soave D. Sun L., 2017 A generalized Levene’s scale test for variance heterogeneity in the presence of sample correlation and group uncertainty. Biometrics 73: 960–971. 10.1111/biom.12651 28099998

[bib36] Sun X. Elston R. Morris N. Zhu X., 2013 What is the significance of difference in phenotypic variability across SNP genotypes? Am. J. Hum. Genet. 93: 390–397. 10.1016/j.ajhg.2013.06.017 23910463 PMC3738833

[bib37] Valdar W. Flint J. Mott R., 2006 Simulating the Collaborative Cross: power of quantitative trait loci detection and mapping resolution in large sets of recombinant inbred strains of mice. Genetics 172: 1783–1797. 10.1534/genetics.104.039313 16361245 PMC1456308

[bib38] Xu S., 1998 Iteratively reweighted least squares mapping of quantitative trait loci. Behav. Genet. 28: 341–355. 10.1023/A:1021617618150 9926616

